# Accordion Maneuver for Delayed Regenerate Formation Following Pediatric Osteosarcoma Resection: A Case Report and Literature Review

**DOI:** 10.3390/reports9030217

**Published:** 2026-07-09

**Authors:** Abdullah Addar, Mishari Alanezi, Nouf Alabdulkarim, Razan Alshatwi, Fahad Alshayhan, Fahad Alhuzaimi

**Affiliations:** 1 Department of Orthopedic Surgery, College of Medicine, King Saud University, Riyadh 11362, Saudi Arabia; 2 College of Medicine, King Saud University, Riyadh 11362, Saudi Arabia

**Keywords:** accordion maneuver, osteosarcoma, bone transport, distraction osteogenesis

## Abstract

**Background and Clinical Significance**: Reconstruction of large segmental bone defects following oncologic resection in pediatric patients remains a major challenge. Although distraction osteogenesis with bone transport is a well-established biological reconstructive option, regenerate formation may be compromised in patients receiving chemotherapy. The accordion maneuver, consisting of alternating cycles of compression and distraction, has been described as a method to stimulate bone regeneration, primarily in association with external fixation systems. However, its use in internal bone transport systems using intramedullary lengthening nails following oncologic resection, particularly in the setting of perioperative chemotherapy, remains rarely reported. **Case Presentation**: We report a case of a 12-year-old boy with high-grade telangiectatic osteosarcoma of the distal femur who underwent neoadjuvant chemotherapy followed by limb-salvage surgery, resulting in a 14 cm segmental bone defect. Reconstruction was performed using plate-assisted bone transport with a motorized intramedullary magnetic nail. During distraction osteogenesis, delayed and asymmetric regenerate formation developed. An accordion maneuver was subsequently initiated, resulting in progressive improvement in regenerate density and corticalization without the need for revision surgery. **Conclusions**: This case highlights the successful application of the accordion maneuver using internal bone transport following oncologic resection. It represents a minimally invasive technique to stimulate bone healing and may reduce the need for revision surgery; however, larger series are needed to confirm this potential benefit, particularly in biologically compromised patients receiving chemotherapy.

## 1. Introduction and Clinical Significance

Osteosarcoma is an aggressive primary malignant bone tumor that typically affects the long bones and requires multimodal treatment, including wide surgical resection [1]. Such resections frequently result in large segmental bone defects, representing a major reconstructive challenge, particularly in pediatric and adolescent patients [2]. Distraction osteogenesis using internal or external fixation has become a well-established technique for managing large bone defects [3]. Bone transport using intramedullary lengthening nails provides better soft tissue tolerance and reduced pin-tract-related complications compared with traditional external fixators [4]. However, regenerate bone formation during distraction osteogenesis can be unpredictable and may be compromised by factors such as chemotherapy, infection, poor biological environment, or altered mechanical stability [5].

The accordion maneuver is a biological stimulation technique used during distraction osteogenesis to enhance regenerative bone formation. It involves alternating cycles of controlled compression and distraction at the osteotomy or regenerate site, aiming to stimulate osteogenesis through mechanical loading and improved vascular response [6].

In this case report, we present a pediatric patient with a large femoral defect following wide resection of a high-grade osteosarcoma in the setting of perioperative chemotherapy. The report highlights the successful application of the accordion maneuver using a fully internal bone transport system to address delayed regenerate formation in a biologically compromised oncologic environment. It focuses on the use of an accordion maneuver as a salvage strategy for delayed regenerate formation during all-internal bone transport and describes the timing, protocol, and short-term radiographic response in this complex oncologic reconstruction. The patient’s guardian voluntarily agreed to publish this report for educational purposes, and informed consent was obtained.

## 2. Case Presentation

**Figure 1:**
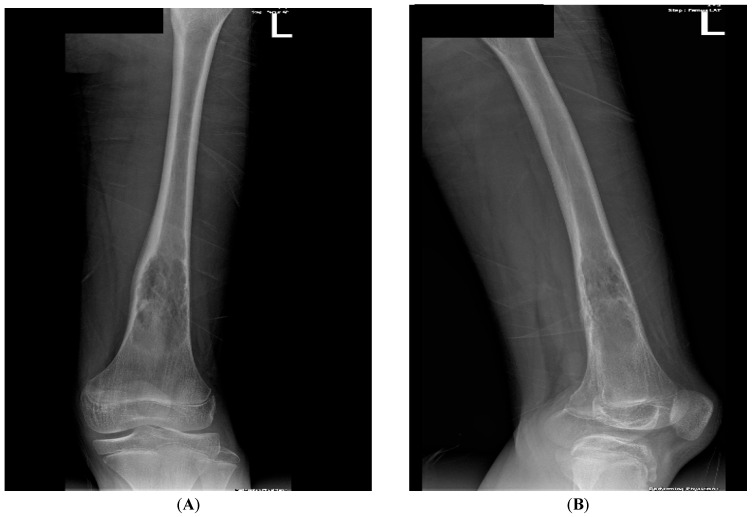
(**A**) Anteroposterior radiograph of the left femur showing an aggressive lytic lesion in the distal femoral diaphysis with cortical destruction. (**B**) Lateral radiograph confirming the extent of the diaphyseal lesion with aggressive radiographic features.

A previously healthy 12-year-old boy presented with progressive left distal thigh pain following a minor traumatic event. The pain gradually worsened over several days and became severe enough to prevent weight-bearing. Initial radiographs revealed an aggressive lytic lesion involving the distal third of the left femoral diaphysis without evidence of fracture (Figure 1).

**Figure 2:**
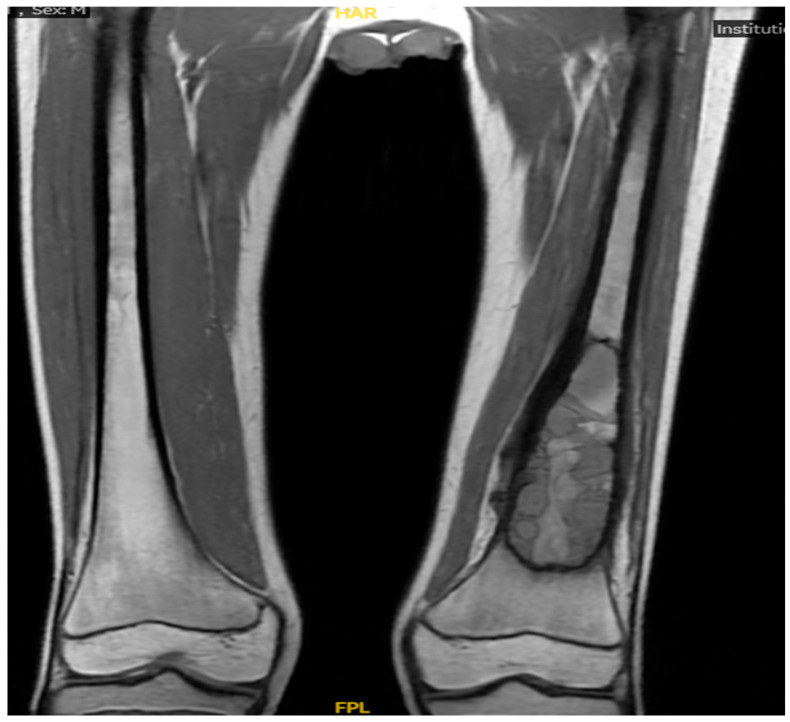
Magnetic resonance imaging of the left femur demonstrating an aggressive intramedullary lesion with cortical breach and soft tissue extension.

Subsequent magnetic resonance imaging (MRI) demonstrated an aggressive intramedullary lesion with cortical destruction and soft tissue extension (Figure 2), raising concern for a primary malignant bone tumor.

Histopathological assessment of the image-guided biopsy specimen confirmed high-grade telangiectatic osteosarcoma. Staging workup, including computed tomography (CT) of the chest and positron emission tomography (PET), showed no definitive evidence of metastatic disease. The patient was discussed at a multidisciplinary tumor board and subsequently received neoadjuvant chemotherapy. Apart from diagnostic biopsy and neoadjuvant chemotherapy, no additional pre-resection local treatment or reconstructive procedure was performed before limb-salvage surgery.

**Figure 3:**
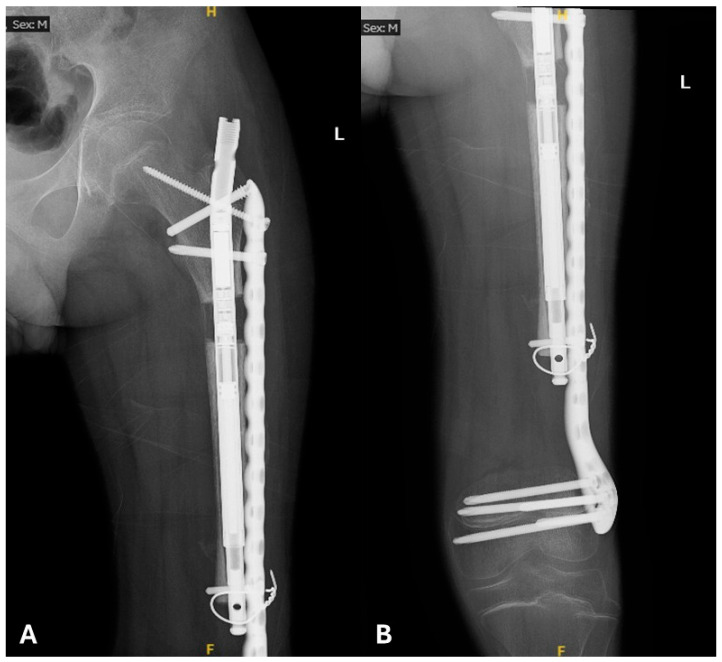
(**A**) Anteroposterior radiograph obtained during distraction osteogenesis demonstrating delayed and asymmetric regenerate bone formation at the transport site, with reduced density along the medial cortex. (**B**) Anteroposterior radiograph illustrating poor regenerate consolidation and nonuniform callus formation during ongoing internal bone transport.

Following completion of neoadjuvant chemotherapy, the patient underwent limb-salvage surgery with wide resection of the distal femur. The oncologic resection resulted in an approximately 14 cm segment defect. Reconstruction was performed using plate-assisted bone transport with a motorized intramedullary PRECICE nail. Because intramedullary nailing was performed during the same operative setting as tumor resection, the osteotomy was created through an open approach. A low-energy technique was used, with multiple drill holes that were subsequently connected and completed using an osteotome to minimize thermal and periosteal injury. After a 7-day latency period, distraction was initiated according to the standard protocol, as adjuvant chemotherapy was not started until 3 weeks after surgery, thereby allowing an initial period of regenerate formation before systemic treatment. The planned transport distance was approximately 14 cm. Distraction was initially performed at a rate of 0.5 mm twice daily under close clinical and radiographic surveillance. However, following the initiation of adjuvant chemotherapy, radiographs demonstrated poor regenerate formation; therefore, the distraction rate was reduced to 0.4 mm/day to optimize regenerate quality. After completion of chemotherapy, the distraction rate was gradually increased to 0.8 mm/day. Despite these adjustments, serial radiographs continued to demonstrate slow and asymmetric regenerate formation, raising concern for delayed consolidation (Figure 3).

Given the patient’s history of chemotherapy and the radiographic appearance of the regenerate, a decision was made to initiate the accordion maneuver using the fully internal motorized intramedullary nail system. The nail was first started in compression mode at a rate of 0.5 mm per day for 7 consecutive days, followed by distraction at the same rate for another 7 days to restore length. This controlled compression–distraction protocol was repeated for two cycles with close weekly radiographic surveillance.

**Figure 4:**
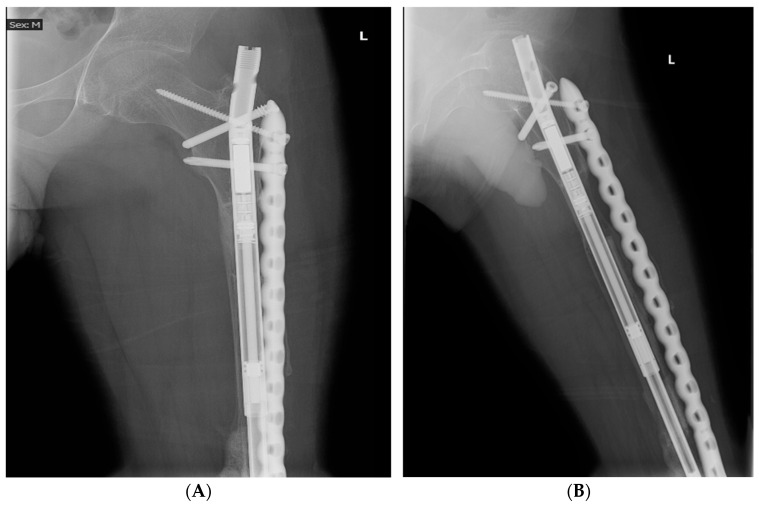
(**A**) Anteroposterior radiograph after initiation of the accordion maneuver showing progressive improvement in regenerate density and early corticalization, particularly along the medial cortex. (**B**) Lateral radiograph showing enhanced regenerate maturation and improved structural continuity following controlled cycles of compression and distraction.

Following implementation of the accordion maneuver, progressive improvement in regenerate density and corticalization was observed, particularly along the medial cortex (Figure 4).

**Figure 5:**
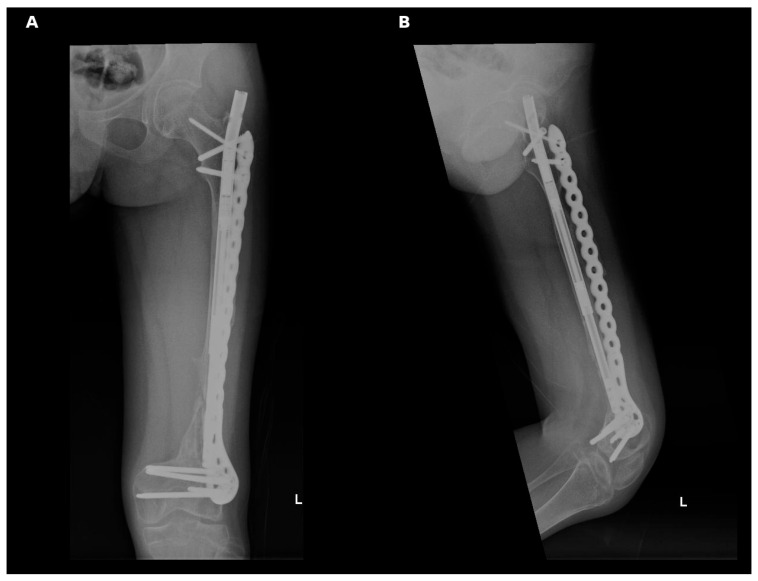
Final follow-up radiographs demonstrating the end result after bone transport and subsequent docking-site reconstruction. (**A**) Anteroposterior radiograph including the adjacent hip and knee joints. (**B**) Lateral radiograph including the adjacent joints.

The patient continued postoperative rehabilitation under protected weight-bearing and received adjuvant chemotherapy as part of the multidisciplinary oncologic treatment plan. Upon completion of transport, direct bone contact and primary union at the distal docking site were not achieved because the intramedullary nail cut through the distal segment before docking was completed. Consequently, a polymethylmethacrylate (PMMA) cement spacer was inserted to maintain the residual defect and induce membrane formation. Six weeks later, the spacer was removed, and the defect was reconstructed with bone grafting using the induced membrane technique. Final radiographs, including the adjacent hip and knee joints, demonstrated radiographic consolidation and union of the reconstructed segment 10 months and 25 days after the index surgery (Figure 5). At the 6-month follow-up, the patient remained clinically stable and neurovascularly intact, with no radiographic evidence of local recurrence. Table 1 summarizes the treatment timelines and radiological follow-up.

## 3. Discussion

Reconstruction of large segmental bone defects following oncologic resection remains a major challenge in pediatric orthopedic oncology. Limb-salvage surgery aims to achieve local tumor control while preserving limb function; however, the resulting bone loss often necessitates complex reconstructive procedures. Distraction osteogenesis and bone transport have become reliable options for managing extensive defects, offering the advantage of biological bone regeneration without the need for massive allografts or endoprosthetic reconstruction. In this context, the present case describes an intramedullary nail-delivered accordion maneuver as a non-operative salvage approach during oncologic bone transport in a child receiving chemotherapy.

Large segmental defects of the long bones have traditionally been managed using several reconstructive techniques, including vascularized fibular grafts, massive structural allografts, endoprosthetic reconstruction, and distraction osteogenesis with bone transport [6,7,8]. Each method carries inherent advantages and limitations. Vascularized fibular grafts provide living bones with an intrinsic blood supply but are associated with donor-site morbidity, prolonged time to hypertrophy, and risk of fracture [6,7]. Massive allografts offer immediate structural stability but carry high rates of nonunion, infection, and graft fracture, particularly in pediatric and oncologic populations [8]. Endoprosthetic reconstruction allows early mobilization; however, it is limited by implant longevity, risk of infection, and the need for revision surgery in growing patients [9]. In contrast, bone transport enables gradual biological reconstruction of large defects, restores limb length, and adapts to growth, making it particularly appealing in pediatric patients [10].

Distraction osteogenesis relies on a delicate balance between mechanical stability and biological healing. However, in oncologic patients, bone regeneration may be adversely affected by systemic factors, most notably chemotherapy [11]. Cytotoxic agents commonly used in osteosarcoma treatment have been shown to impair osteoblast proliferation, inhibit angiogenesis, and disrupt callus formation, leading to delayed consolidation or poor regenerate quality during distraction osteogenesis [12]. Additionally, chemotherapy-induced anemia, nutritional compromise, and altered inflammatory response may further negatively impact bone healing [13]. These biological challenges necessitate close monitoring of regenerate formation and early intervention when delayed or asymmetric regenerate is identified.

The accordion maneuver is a mechanical modulation technique used during distraction osteogenesis to stimulate bone regeneration in cases of poor regenerate formation. It involves alternating cycles of controlled compression and distraction at the regenerate site, typically applied when radiographic evidence suggests delayed consolidation or inadequate callus formation [14]. The proposed mechanism involves enhanced mechanotransduction, increased local vascularity, stimulation of osteogenic differentiation, and improved maturation of regenerate bone through cyclic loading [15]. It has been most extensively described in the context of external fixation systems, particularly circular frames, where it has been shown to improve regenerate quality and reduce the risk of nonunion [14,16]. With external fixators, the accordion maneuver is most commonly performed using daily modulation protocols, typically involving small incremental adjustments (commonly 0.25–1.0 mm/day in two or more steps) [17]. Some reported regimens apply daily compression at 0.5 mm/day followed by slower distraction (e.g., 0.25 mm/day), whereas others reverse the sequence or use different rhythms, including weekly phase changes [14]. For instance, Makhdom et al. reported a series of four tibial lengthening cases with absent or delayed regenerate formation. A three-step daily sequence (0.25 mm distraction, 0.25 mm compression, followed by 0.25 mm distraction) resulted in a net gain of 0.25 mm/day and was applied over several weeks [14]. Radiographic improvement and progression to consolidation were observed in three cases, while one case required additional intervention due to suspected infection [14]. In contrast, reports describing the use of the accordion maneuver within fully internal bone transport systems remain limited. Recent preclinical evidence from Bertrand et al. reported that accordion protocols did not improve bone healing in a murine distraction osteogenesis model [18]. However, extrapolation from rodent models to pediatric oncologic patients is limited by differences in bone biology, regenerate volume, mechanical environment, and systemic effects of chemotherapy. Murine models lack the prolonged latency, large segmental defects, and complex mechanobiology present in human femoral bone transport. In contrast, clinical reports using circular external fixators and the present case using an internal lengthening nail suggest that cyclic compression–distraction may enhance mechanotransduction and vascular ingrowth in large, biologically compromised human regenerates. These discrepancies highlight the need for caution when interpreting animal data and support further clinical investigation of accordion protocols in human bone transport, particularly with internal devices. No standardized protocols have been clearly established for the application of the accordion maneuver using intramedullary lengthening nails. Unlike external fixators, internal motorized nails require device reprogramming and may present practical challenges for frequent alternation between compression and distraction. Accordingly, a weekly alternation strategy (7 days of compression followed by 7 days of distraction) was used in the present case. This approach aimed to balance biological stimulation with mechanical practicality while maintaining close radiographic monitoring of regenerate response.

In the present case, the accordion maneuver was applied in response to delayed and asymmetric regenerate formation using an internal bone transport following a distal femoral osteosarcoma resection. After initiation of controlled compression–distraction cycles, radiographic improvement in regenerate density and corticalization was observed, supporting the applicability of this technique even in a biologically compromised environment. This suggests that the accordion maneuver can serve as an effective salvage strategy during internal bone transport without the need for surgical revision or alteration of the reconstructive construct. Final radiographic consolidation/union was achieved 10 months and 25 days after the index surgery; however, direct distal docking-site contact was not achieved, and salvage with PMMA spacer insertion followed by induced membrane bone grafting was required. Therefore, the case supports the technical feasibility of intramedullary nail-delivered compression–distraction for poor regenerate quality but cannot be interpreted as standalone proof of successful docking-site reconstruction.

Despite its benefits, the accordion maneuver is not without potential risks. Excessive compression may lead to regenerate collapse, pain, or mechanical instability, while inappropriate distraction parameters may further impair bone healing [17]. Therefore, careful patient selection, close radiographic monitoring, and individualized adjustment of compression–distraction cycles are essential to minimize complications and optimize outcomes.

This case contributes a context-specific observation regarding mechanical modulation for poor regenerate formation during all-internal bone transport. Previous reports have described accordion maneuvers using magnetically controlled intramedullary lengthening nails, primarily in non-oncologic limb-lengthening settings or traumatic bone defects. Extension of this approach to pediatric oncologic reconstruction may be clinically relevant given the biologically compromised environment associated with perioperative chemotherapy. Further studies are required to better define indications, nail-specific protocols (including phase duration and total excursion), and objective clinical and radiographic outcomes for this adjunctive technique during internal bone transport.

## 4. Conclusions

Large segmental bone defects following oncologic resection in pediatric patients remain challenging to reconstruct, particularly in the setting of chemotherapy-related impairment of bone healing. This case demonstrates the technical feasibility of using an intramedullary magnetic lengthening nail to deliver an accordion maneuver during plate-assisted internal bone transport, with associated radiographic improvement in regenerate quality. This technique may reduce the need for revision surgery; however, larger series are needed to confirm this potential benefit.

## Figures and Tables

**Table 1:** Treatment timeline and key radiological findings during plate-assisted internal bone transport and regenerate salvage.

Time Point	Management Step	Key Radiological Finding or Interpretation
Presentation and staging	Radiographs, MRI, image-guided biopsy, CT chest, and PET staging were performed.	Aggressive distal femoral lesion with cortical destruction and soft-tissue extension; high-grade telangiectatic osteosarcoma was confirmed, with no definitive metastatic disease.
After neoadjuvant chemotherapy/index surgery	Wide distal femoral resection and plate-assisted internal bone transport with a motorized intramedullary PRECICE nail were performed.	The post-resection segmental defect measured approximately 14 cm. Accordingly, the planned transport distance was 14 cm to achieve complete reconstruction of the defect.
Postoperative day 7	Distraction was initiated at a rate of 0.5 mm twice daily	Distraction was started before adjuvant chemotherapy to allow early regenerate formation.
3 weeks postoperatively and during adjuvant chemotherapy	Adjuvant chemotherapy was commenced; the distraction rate was reduced to 0.4 mm/day.	Relatively poor regenerate formation was observed radiographically during systemic treatment.
After chemotherapy completion	The distraction rate was gradually increased to 0.8 mm/day.	Regenerate formation improved radiographically after chemotherapy completion.
During distraction after delayed/asymmetric regenerate formation	The accordion maneuver was delivered through the intramedullary nail using controlled compression and distraction cycles.	Progressive improvement in regenerate density and corticalization was observed, particularly along the medial cortex.
Before distal docking completion	Direct docking-site contact was not achieved after nail cut-through of the distal segment; a PMMA cement spacer was inserted.	Primary distal docking-site union was not achieved during the initial transport phase.
Six weeks after spacer insertion	Spacer removal and bone grafting using the induced membrane technique were performed.	The residual defect was reconstructed biologically after membrane formation.
10 months and 25 days after index surgery	Final AP and lateral radiographs, including adjacent joints, were obtained.	Radiographic consolidation/union of the reconstructed segment was demonstrated after additional docking-site management.

AP, anteroposterior; CT, computed tomography; MRI, magnetic resonance imaging; PET, positron emission tomography; PMMA, polymethylmethacrylate. Index surgery refers to wide distal femoral resection and reconstruction with plate-assisted internal bone transport using a motorized intramedullary nail.

## Data Availability

The original contributions presented in this study are included in the article. Further inquiries can be directed to the corresponding author.

## Abbreviations

The following abbreviations are used in this manuscript:

CT	Computed tomography
MRI	Magnetic resonance imaging
PET	Positron emission tomography
PMMA	Polymethylmethacrylate
